# Operational challenges in the pre-intervention phase of a mental health trial in rural India: reflections from SMART Mental Health

**DOI:** 10.1186/s13033-022-00549-4

**Published:** 2022-08-16

**Authors:** Ankita Mukherjee, Mercian Daniel, Amanpreet Kaur, Siddhardha Devarapalli, Sudha Kallakuri, Beverley Essue, Usha Raman, Graham Thornicroft, Shekhar Saxena, David Peiris, Pallab K. Maulik

**Affiliations:** 1 The George Institute for Global Health, New Delhi, India; 2 The George Institute for Global Health, Hyderabad, India; 3 The George Institute for Global Health, UNSW Sydney, Sydney, Australia; 4 University of New South Wales, Sydney, Australia; 5 Institute for Health Policy, Management and Evaluation, University of Toronto, Toronto, Canada; 6 Department of Communication, University of Hyderabad, Hyderabad, India; 7 Institute of Psychiatry, Psychology and Neuroscience, King’s College London, London, UK; 8 Harvard T H Chan School of Public Health, Boston, USA

**Keywords:** cRCT, Operational challenges, LMIC, Mental Health Services, Rural, India, Complex intervention, mHealth

## Abstract

**Background:**

Availability of mental health services in low- and middle-income countries is largely concentrated in tertiary care with limited resources and scarcity of trained professionals at the primary care level. SMART Mental Health is a strategy that combines a community anti-stigma campaign with a primary health care workforce strengthening initiative, using electronic decision support with the goal of better identifying and supporting people with common mental disorders in India.

**Methods:**

We describe the challenges faced and lessons learnt during the *pre-intervention phase* of SMART Mental Health cluster Randomised Controlled Trial. Pre-intervention phase includes preliminary activities for setting-up the trial and research activities prior to delivery of the intervention. Field notes from project site visit, project team meetings and detailed follow-up discussions with members of the project team were used to document operational challenges and strategies adopted to overcome them. The socio-ecological model was used as the analytical framework to organise the findings.

**Results:**

Key challenges included delays in government approvals, addressing community health worker needs, and building trust in the community. These were addressed through continuous communication, leveraging support of relevant stakeholders, and addressing concerns of community health workers and community. Issues related to use of digital platform for data collection were addressed by a dedicated technical support team. The COVID-19 pandemic and political unrest led to significant and unexpected challenges requiring important adaptations to successfully implement the project.

**Conclusion:**

Setting up of this trial has posed challenges at a combination of community, health system and broader socio-political levels. Successful mitigating strategies to overcome these challenges must be innovative, timely and flexibly delivered according to local context. Systematic ongoing documentation of field-level challenges and subsequent adaptations can help optimise implementation processes and support high quality trials.

*Trial registration*: The trial is registered with Clinical Trials Registry India (CTRI/2018/08/015355). Registered on 16th August 2018. http://ctri.nic.in/Clinicaltrials/showallp.php?mid1=23254&EncHid=&userName=CTRI/2018/08/015355

## Background

Low and middle-income countries (LMICs) experience a large disease burden from mental disorders [1, 2]. Only a small proportion of individuals needing mental health care can access it, leading to large treatment gaps [3, 4]. In India, around 150 million people are in need of care for depression, anxiety, and alcohol and other substance use disorders [5]. Major barriers to accessing mental health services include the stigma associated with mental illnesses and care seeking, lack of a trained workforce especially in primary care settings, limited public funding for mental health services and large out-of-pocket costs [2, 6–8].

The Systematic Medical Appraisal Referral and Treatment (SMART) Mental Health programme aims to reduce treatment gaps for psychological stress, depression, anxiety and increased self-harm risk (collectively referred to here as common mental disorders for the project) in rural India [9]. The integrative strategy comprises of two broad components—a community level anti-stigma campaign to address community demand side barriers and the use of an electronic decision support system (EDSS) using mobile devices to help community health workers and primary health centre (PHC) doctors in the diagnosis and management of people with common mental disorders (CMDs).

Previous studies have identified several challenges in implementation of complex interventions including demands on staff, patient preferences, patient-staff dynamics, difficulties in recruitment and retention in trials, and organisational context. Additionally, external factors including policy or market level changes may also impact such interventions [10–12]. These challenges have been reported in both hospital-based trials as well as community-based trials, mainly from high income country settings with limited literature on community-based trials in LMICs [13].

**Fig. 1:**
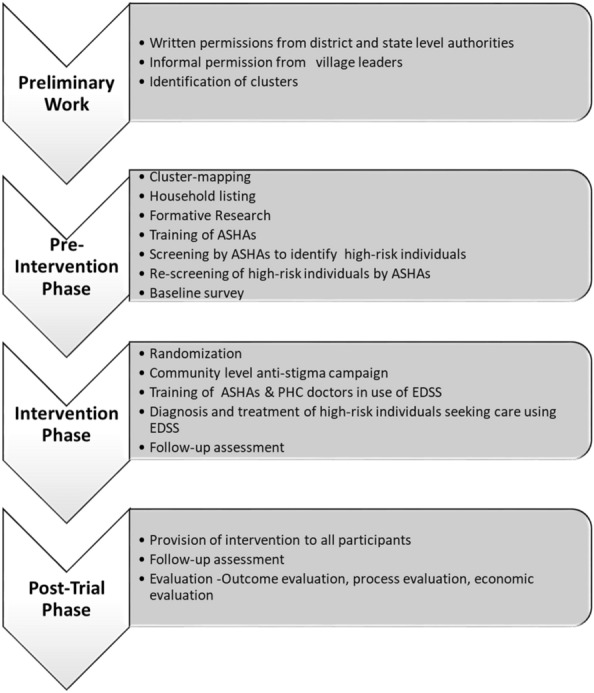
SMART Mental Health-Project Phases

SMART Mental Health is being evaluated in a cluster randomized controlled trial (cRCT) with specific activities in the pre-intervention, intervention, and post-intervention phases (Fig. 1). This paper focuses only on the pre-intervention phase of the SMART Mental Health project. It aims to identify contextual factors that may influence operationalising complex interventions, provide information about adaptations which need to be made to address emergent challenges, and address knowledge gaps on implementation barriers of health service interventions in LMICs [14].

## Method

### Project background

SMART Mental Health is being implemented in three districts across two states in India: West Godavari district in the southern state of Andhra Pradesh, Palwal and Faridabad districts in the northern state of Haryana. West Godavari is a coastal district with a population of about 3.9 million [15]. Telugu is the most commonly spoken language in the area. Palwal and Faridabad districts are close to the national capital New Delhi. Faridabad with a population of about 1.8 million [16] is nearly 45 kms m from New Delhi while Palwal with a population of close to 1 million [17] is nearly 75 kms from the national capital. Hindi and the local dialect Haryanvi are the most spoken languages in these districts. All the three districts are predominantly rural agricultural areas. The districts have differences in availability of mental health services in government settings. Faridabad district has one psychiatrist at the district hospital as well as out-patient psychiatry services available at another government hospital. Palwal district hospital does not have a psychiatrist. The psychiatrist from Faridabad district hospital provides services in Palwal once a week. West Godavari has a well-functioning psychiatry unit at the district hospital. The psychiatrist also makes regular visits to the PHCs in her district to offers psychiatry out-patient services on specified days. Private psychiatric services are available more widely in West Godavari and Faridabad compared to Palwal.

A cluster for  SMART Mental Health cRCT is the Primary Health Care Centre (PHC) and the communities serviced by that PHC. A rural PHC in India typically serves a population of about 30,000 people with one at least one medical doctor available in each PHC [18] supported by other paramedical and administrative staff. The PHCs are the lowest rung of the primary health care delivery system which has a doctor.

The *pre-intervention phase* consists of preliminary groundwork for setting up the study and research activities prior to randomization of clusters and delivery of the intervention components. This included identification of PHC services, obtaining permissions from state and district level health authorities and consultation with village stakeholders including elected representatives. Research activities included:

(i) site visits to draw maps of clusters with relevant information (such as location of health centres, pharmacies);
(ii) village level census for listing of all households in the study clusters;
(iii) formative research to obtain feedback from community and health workers on the proposed intervention tools (app based EDSS and anti-stigma campaign content);
(iv) training of community health workers known as Accredited Social Health Activists (ASHAs) in India to screen for CMDs;
(v) household screening undertaken by ASHAs with standardised screening tools—Generalized Anxiety Disorder-7 item(GAD-7) [19, 20] and Patient Health Questionnaire-9 item scales(PHQ-9) [21, 22]—using handheld tablet devices,to identify individuals at high risk of common mental disorders;
(vi) a confirmatory re-screen of people identified at high-risk cohort within 6 months of initial screening (to exclude those who have experienced natural remission); and
(vii) a baseline survey by an independent data collection team, to measure knowledge, attitude and practices related to mental health.

In the *intervention phase* of the trial, those PHCs allocated to SMART health will receive a community level anti-stigma campaign using print and audio-visual communication tools and enhanced mental health service delivery by PHC doctors and ASHAs using EDSS. The EDSS uses World Health Organization’s Mental Health Gap Action Programme- Intervention Guide (mhGAP-IG) to support diagnosis and treatment decisions of PHC doctors. It provides ASHAs information on high-risk persons who need to be followed-up and referred to the PHC doctors. PHCs in the control arm will receive enhanced usual care in which all individuals identified at high risk of a CMD will be encouraged to attend their local PHC and/or seek specialist opinion. The intervention will be provided for 12 months. Following this in a *post-trial phase* the intervention components will be provided to both usual care and intervention arms with minimal support from research staff to assess the sustainability and scalability of the intervention [9].

### Identifying challenges

To identify the key challenges faced during the pre-intervention phase, field notes from the following sources were used:

- Observation during visits to the project site (in Haryana only)
- Discussion with ASHAs and field staff during field visits in Haryana and using online modes in Andhra Pradesh
- Meeting notes involving research fellows at both sites
- Notes from weekly project update meetings with the principal investigator
- Email correspondence with team members

All weekly team meetings of the research fellows with the principal investigator were attended by the author (AM). Due to COVID restrictions, field visits could not be made to Andhra Pradesh. A review of notes from meetings and field visits helped in identifying a broad range of operational challenges faced in the pre-intervention phase. To gain greater insights into these challenges, discussion with eight project team members (Table 1) were held. Domains covered during these discussions are indicated under the ‘challenges’ column in Table 2. The project team members were asked to discuss in detail about each of the challenges identified in the field notes, strategies used to overcome them and any lessons they felt were learnt. They were also asked to share any operational challenges that had not been captured in the field notes. These discussions were held in-person, telephonically, through video chat, over email and included inputs from the research fellows, IT staff, and field level project supervisors in both states. Two research fellows and one field supervisor were included from Andhra Pradesh site while two research fellows and two field supervisors were included from Haryana site, apart from the IT staff working for both sites. Information provided by project team supplemented the field notes and provided a detailed picture of challenges during the pre-intervention phase and strategies adopted by the team to overcome these challenges. No formal audio recording of the discussions with team members were done. However written responses from email communication have been quoted here in the paper.

**Table 1:** SMART Mental Health project team members with whom discussions were held

Position	Number	Role
Research Fellows	4	Planning and monitoring of research activities
Project Supervisors	3	Supervision of enumerators and executing all field level research activities
IT Staff	1	App development and providing tech-enabled solutions
Total	8

**Table 2:** Key Operational Challenges in the pre-intervention phase and strategies used to overcome them

No	Challenges faced	Details	Strategies implemented to overcome the challenges
I	*Individual Level*		
1	Low motivation of some ASHAs to participate in the project	Some ASHAs were not motivated to be part of the project. They felt involvement in the project had led to an increase in their work burden In AP, due to significant increase in remuneration of ASHAs, several of them did not find additional income from the project as an attractive incentive and were unwilling to take on project related tasks in addition to their regular work Some resorted to avoiding contact with field staff and tried to postpone screening individuals’ multiple times	Persons senior to ASHAs, who were likely to convince ASHAs to be part of the project were approached. These included the PHC medical officers, District and Block ASHA co-ordinators. They explained to the ASHAs that participating in the project would equip them for government mental health programmes in the future and was a good opportunity to learn using tablets In some places ASHAs were replaced with other local women with similar educational background. They were trained and provided support by field staff for the data collection
	Unsupportive attitude of some stakeholders	In one site an ASHA facilitator asked all ASHAs in her locality to stop working on the project because she was dissatisfied with the amount being paid by the project In one site some Auxiliary Nurse Midwife (ANMs) were not happy with ASHA’s involvement in the project. ASHAs report to ANMs and work closely with them. Some ASHAs wanted to withdraw from the project since they did not want to displease the ANM	Persons senior to ASHAs were approached. They were requested to convince ASHAs to work in the project The medical officers of PHCs where the ANMs worked were approached. They were requested to call ANMs and convey the need to provide support to the project
	Dissatisfaction related to remuneration among some ASHAs	Several ASHAs in Haryana were dissatisfied with the incentive amount offered to them to undertake screening of high-risk individuals In Andhra Pradesh, there was a significant hike in salaries being paid to ASHAs by the government. Remuneration for time through the project was small compared to their monthly salary. Therefore, many ASHAs were not motivated to work for the project and opted out	Meetings were held with ASHAs to address their concerns. It was explained that fee being paid to them was at par with existing government rates for similar tasks. Details of how the time commitment and remuneration were at par with government rates was discussed Field staff approached the PHC Medical Officers on specified days when ASHAs came to the PHC for their weekly meeting. They were requested to talk to ASHAs and convince them
	Low performance by ASHAs in some clusters	There was variation in performance of ASHAS in different clusters. In some clusters ASHAs took much longer than expected, to complete screening of high-risk individuals	A teleconference was organised for ASHAs of high performing and low performing clusters. This provided them an opportunity to interact and learn from their peers and motivated ASHAs of low performing clusters to improve
	Reaching all individuals in the sample	In Haryana the sample for high-risk screening was randomly selected. Many individuals lived in areas far from the ASHAs home. As majority of ASHAs did not have private means of transport and found it difficult to collect data Some individuals selected in the sample were migrants who were not available when the ASHA visited them	Regular contact with ASHAs and on-site support for any difficulties arising in the field was provided by project staff to ensure that all individuals in the sample were reached by the ASHA Where possible the ASHAs travelled with the field staff on their motorbikes to respondent households
II	*Interpersonal Level*		
1	Objections by family members of some ASHAs	In Haryana, due to the cultural context very few female field staff were recruited. Therefore, male staff were required to work with ASHAs. Several ASHAs were reluctant to move around the village with male field supervisors. Family members of some ASHAs expressed displeasure at having male staff travel with ASHAs	Male staff were sensitive to adopt culturally appropriate behaviour in their interaction with ASHAs. Senior field supervisors reached out to family members and explained the project goals and need for supervision
2	Creation of anti-stigma video of person with lived experience (PWLE)	One of the campaign materials was a video of a PWLE of mental illness, sharing his/her story. In Haryana it was not possible to find any PWLE who was willing to share his/her lived experience on camera. Family members objected due to fear of stigma from relatives and neighbours	The video from Andhra Pradesh site was dubbed in local language and used in Haryana
III	*Institutional/Organisational Level*		
1	Approvals and permissions	Getting permissions before the start of the project was a long-drawn process and took more time than was anticipated. Permissions were taken from state level health directorate and the district level	Repeated visits were made by the project staff to concerned officials Other stakeholders who could discuss the project with concerned officials (like elected representatives), were approached for their support
2	Transfer of key health officials	Before start of the project, efforts were made to explain project objectives and have buy-in from district level health officials. In one site the health official who had been approached at the start of the project was transferred Some planned trainings with ASHAs were delayed because the district health official was transferred, and permissions needed to be taken from the new appointee	Senior project staff visited the newly appointed official and shared relevant information about the project and prior permissions that had been granted
3	Delay in communication between departments	There was delay in sharing information about the project to the PHC doctors by district level authorities. Without formal communication from the district level, PHC doctors were unwilling to permit ASHAs to participate in the project In Haryana additional permissions were needed to conduct training of ASHAs. PHC doctors needed office orders issued from the CMOs office before allowing trainings	Contact was made with PHC doctors in all the PHC clusters by project staff. The project objectives were explained, and their co-operation requested Copies of formal permission letters from the state and district level authorities were shared with the PHC doctors Senior project staff met the CMOs, nodal program in-charge, and district ASHA co-ordinators to seek permission and get the office order issued
4	Selection and mapping of clusters	For selection of PHC clusters several inclusion and exclusion criteria (eg. rural PHCs, contiguity) needed to be considered. However, obtaining PHC level data was a challenge Obtaining official map for districts in Haryana was a challenge	All PHCs were physically visited by staff to verify they met the inclusion criteria Several sources were explored to obtain district map and other relevant data (eg. district handbooks, census data, surveys done by ASHAs, and municipal corporation)
	Time spent making changes in the apps	Any existing modifications of the applications needed to be done manually	Remote online modifications of applications were introduced
	Data Safety	Family members of some ASHAs were able to bypass the AppLock feature and were misusing the tablet for downloading videos	More stringent data safety measures were put in place ASHAs were informed about the importance of not using the tabs for personal downloads as it could compromise the functionality of the tabs Additional data encryption measures were added so to ensure data safety in case of the tab being lost or stolen
IV	*Community Level*		
1	Winning community support and trust	In Haryana the intervention area was new for the implementing agency. During household listing, field workers noted distrustful attitude in some villages. Some villagers had past negative experience with other NGOs. Some villagers thought that field enumerators could be thieves gathering household information	Relevant information about the organisation, office location and contact details of supervisory staff who villagers could contact for more information were shared Local staff who belonged to the same area proved helpful in building initial rapport and trust with the community Key village administrators and village leaders including religious leaders contacted wherever needed to discuss the project objectives
2	Socio-cultural norms	In both states ASHAs belonging to Scheduled Caste community were hesitant to go to homes of non-Scheduled caste and talk to them. (Scheduled Castes are castes recognised as socially vulnerable and provided constitutional protection [55]	ASHAs were counselled about the importance of their role and the need to reach out to all households. Field staff accompanied them to such households
3	Mental Health Stigma	During rescreening of high-risk individuals, several individuals expressed displeasure at having a repeat visit from the project staff. Due to the stigma associated with mental health, they feared being labelled as someone with mental health issues in their village	Clear information was provided in the informed consent forms about the nature of the study and possibility of repeated visits to the same households It was explained that primary purpose of the project was to gather information in the initial stage. However, in the later phase of the project some village level medical camps would be organised, and efforts will be made to make medicines available at the PHC
4	Explaining technicalities of research design to community members	During data collection, it proved difficult to explain the research methodology to the community. Some individuals who were selected as part of the random sampling wanted to know why they were being screened and not everyone in their village. They were afraid of being stigmatised as someone with mental health issues The study required repeated visits to the community respondents (screening, re-screening, baseline survey). Several respondents expressed displeasure at having repeated visits There was expectation of some form of benefits from the project like free medicines	All efforts were made to provide information about the research, explain its objectives and selection criteria. Project team used simple and culturally relevant analogies to explain simple random sampling technique, i.e. while steaming rice, to check if it is cooked, only a small portion is checked
V	*Policy/Environment Level*		
1	Protests against the Citizenship Amendment Act (CAA) and National Register of Citizens (NRC)	Challenges were also faced during the anti CAA-NRC protests in early 2020. Due to mistrust for the government, members from the Muslim community in some villages did not want to sign any official looking documents. The villagers were distrustful of signing the information consent form and suspected that the field workers were from a government department Despite taking permission from local village leaders, field workers in Haryana who were collecting data for the baseline survey faced refusal and even verbal and physical threats in several villages There were similar refusals in some villages in Andhra Pradesh	Help was solicited from elected village representatives (*Sarpanch).* The objective of the research, the need for signing the informed consent form and detailed information about the implementing agency was provided. In some cases, the village *sarpanch* accompanied the enumerators or sent his representatives to convince the villagers who were distrustful of the data collection process The local religious leader (*maulvi*) in some villages were approached to solicit co-operation
2	COVID Pandemic	The pandemic and resulting lockdown from March 2020-May 2020 led to delays in the project In Andhra Pradesh where screening of high-risk individuals was resumed after easing of restrictions, villagers expressed displeasure at having outsiders (project staff) come to their village. This made it difficult to supervise work of ASHAs In AP training of staff for baseline and training of ASHAs for rescreening could not be organised face to face due to COVID restrictions	To counter the unpredictability due to COVID, the project team developed three alternative scenarios with three different timelines (Plan A, Plan B, Plan C) with necessary adaptations Risk assessments done periodically at project and at institutional level Monitoring was done through telephonic follow-up with ASHAs. Any missing data or suspected discrepancy in data was checked by calling up the respondent The field teams monitored the official COVID risk classification in the project area and resumed work in designated green zones after the lockdown Online training was organised with small batches spread over a larger number of days than planned earlier To maintain regular communication and monitor work of field staff, a group calling plan was acquired by supervising staff. Regular meetings were held using group calls
3	Strike by ASHAs	The pre-intervention training was planned for the ASHAs in Haryana was delayed because the ASHAs went on a strike due to grievances against existing government norms	Regular communication was maintained with the ASHAs and they were requested to participate in the trainings as soon as they could

### Analysis

**Fig. 2:**
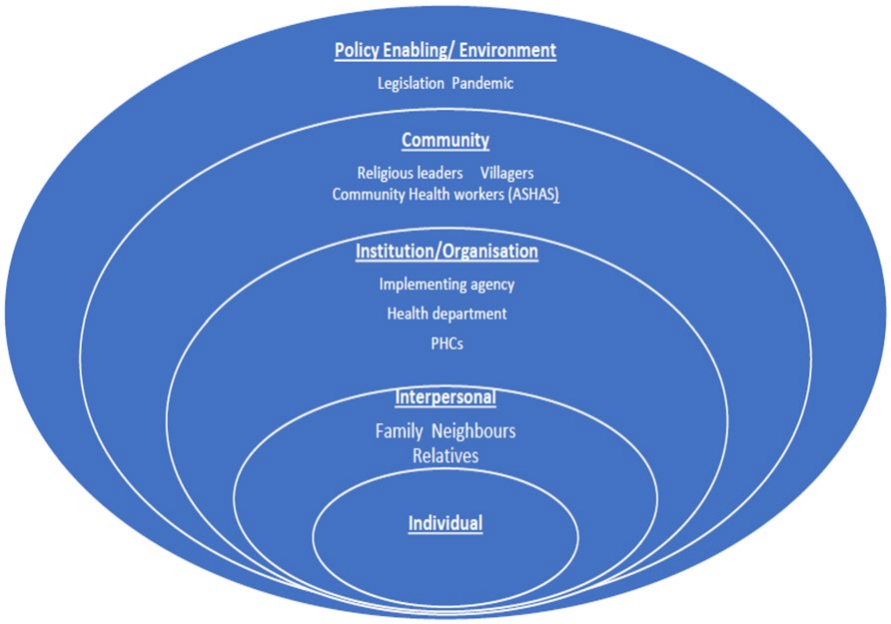
Socio-Ecological Model Levels at which operational challenges were faced and actions were taken in the SMART Mental Health cRCT

The above data sources were reviewed to identify key areas where challenges were faced during the pre-intervention phase. It was felt that a framework for organising all the challenges listed by the project team would provide better understanding of the findings. The Socio-ecological Model (SEM) [23] was used to organise identified challenges, based on levels where these challenges emerged. This was reviewed by the project team to arrive at a consensus. The SEM uses nested concentric circles to situate micro- to macro-level factors that impact a problem (Fig. 2). It identifies various levels—individual, interpersonal, institutional/organisational, community and environment—at which factors impacting a problem may be located. This framework has been used widely to understand factors or determinants at various levels which influence a problem [24], to design multi-level interventions [25] and as a framework for evaluation of intervention strategies [26].

### Ethics

The study received ethics approval from the Independent Ethics Committee of the George Institute, India and the All India Institute of Medical Sciences, New Delhi. It has been registered with Clinical Trials Registry India (CTRI/2018/08/015355).

## Results

During the pre-intervention phase, several challenges were faced at different levels, and these are summarized in Table 2. Below we elaborate on the key issues identified at each level. Many of the challenges and strategies adopted to address them corresponded to multiple levels.

### Individual level

While engaging with ASHAs, one major challenge related to dissatisfaction amongst some ASHAs with the remuneration they were receiving for their involvement in the project, which impacted their motivation to participate in the project.

*“During the screening phase ASHAs in some PHCs had stopped working and had started influencing ASHAs in other PHCs to stop working too, which also coincided with state union strikes. This would have greatly jeopardised completing screening activities on time and would have delayed future activities too”. (Research Fellow 1, Haryana).*

Strategies to address this included open dialogue with ASHAs to understand their grievances and talking to relevant line managers connected to ASHAs. A revised incentive structure was put in place to address remuneration concerns.

*“We spoke with the concerned ASHAs who were not willing to work and tried to understand their concerns. We then revised the screening incentive structure, i.e., from payment being capped for a particular number of screenings to (payment) per screening completed” (Research Fellow 1, Haryana).*

Building relationships with ASHAs was considered critical to sustaining their involvement in the programme *“With ASHAs the work seems routine, but they have different issues at individual and group levels. Building a rapport with the ASHAs and talking to them regularly formally and informally, having regular meetings and discussions with them resolved many issues. (Research Fellow 2, Andhra Pradesh).*

There were some differences in the two states in terms of willingness of ASHAs to participate in the project. In AP we had to replace nearly 15% of the ASHAs with local staff in the pre-intervention phase. While some dropped out due to personal reasons, others cited high work burden. Another reason stated was the significant increase in remuneration of ASHAs in AP. Several ASHAs did not feel the need for additional monetary support that the project provided. In contrast only about 5% ASHAs had to be replaced in Haryana.

### Interpersonal level

Some challenges at the level of the family were also identified. Family members of some ASHAs in Haryana were unhappy for them being accompanied by male field staff in the village. Senior supervisory staff reached out to family members, listened to the concerns raised by them and tried to address these by providing detailed information on the project, its goals, and the need for supervision. The family were assured that male staff would always follow culturally appropriate behaviour. This was a challenge specific to Haryana, as norms around segregation between men and women (especially younger women) are quite marked there compared to Andhra Pradesh. Another challenge in Haryana was finding persons with lived experience (PWLE) of mental illness who were willing to share their experience on camera as part of the anti-stigma campaign materials. Concerns from family members due to fear of stigma was an important reason behind refusal from PWLEs.

*““As part of screening phase in rural Haryana, some ASHAs and field staff did manage to identify few PWLEs who were ready to share their stories with the research and field team. However, they refused to give consent for the same given their family’s apprehensions regarding their reputation in society, future marriage alliances and being labelled with stigmatized words and adjectives.” (Research Fellow 3, Haryana).*

Despite sustained efforts to find PWLEs willing to be featured in the anti-stigma videos in Haryana, the plan ultimately had to be abandoned. Instead, videos from consenting PWLEs from the other study region (Andhra Pradesh) were translated and dubbed into the local language and dialect of Haryana. Additional time was taken in obtaining consent from individuals featured in the videos from Andhra Pradesh and in addition to the translation process took much longer than was originally anticipated.

### Institutional/Organisational level

Many of the challenges were faced at the institutional/organisational level. Some of these were related to external institutions like the health department. Challenges included delays in obtaining approvals/permissions, transfer of officials, and delays in written communication between the higher and lower levels of the concerned department.

Some challenges needed to be addressed by the project team at the internal organisational level. For example, challenges related to technical aspects of the mHealth platform, which required iterative changes to be made by the informational technology support team. There were some difficulties experienced by ASHAs related to the user-interface which was resolved by adopting appropriate solutions to make the app more user-friendly for ASHAs. Based on continuous feedback from the project team, several changes were also made to improve data safety and security, data analytics, and improved backend processes (discussed in Table 2).

### Community level

Issues related to mistrust, prevailing sociocultural norms/beliefs, stigma related to mental health, were emerged at the community level in both sites. This required continuous engagement with the community by the project team to build trust and included enlisting support of local leaders including village elders, community leaders, local administrators, and religious leaders. Political unrest due to community opposition to two citizenship legislations—the Citizenship Amendment Act and the National Register of Citizens—emerged as an unanticipated challenge, which needed to be addressed at the community level. Although the challenge emerged due to change in policy context, the implications were at the community level. The two laws had implications for sharing of personal identifiers for official purposes, which led to subsequent mistrust by religious minorities from some villages to offer any data or sign any documents such as research consent forms. Due to mistrust of the government, any outsiders who came to the village to gather any form of information were viewed suspiciously. Despite taking permission from local village leaders, field workers who were collecting data for the baseline survey faced refusal and even verbal threats in several villages in both Haryana and Andhra Pradesh.

*“There were bigger challenges when the whole community was opposed to (us) collecting information and the issue went on till the staff received a warning of dire consequences. Considering it as a refusal at a community level, efforts were made to meet (persons who had refused) again and again. This did not work at all as the entry itself was closed and (we) couldn’t have a dialogue, not even with a single person from the community.” (Research Fellow 2, Andhra Pradesh).*

To address these concerns, efforts were made to solicit support from influential persons including religious leaders, administrators, elected local body representatives, village elders, and influential community leaders from other villages and political party leaders. In Andhra Pradesh a breakthrough came after help was sought from the religious leader of a nearby village. Project team members reached out to the religious leader and explained the purpose of data collection, the overall goal of the project and provided information about the research institute and clarified any doubts he had. He was aware of some of the work done in the area by the project team in the past and was appreciative of the institute’s efforts to address mental health issues in the area. He reached out to several members of his community including the community head of the village and discussed with them the purpose of the research, the concerns about data sharing, and clarifyied the distinction between the policies of the national government and the activities of the research team.

### Policy/enabling environment

The COVID-19 pandemic was a significant environmental level challenge which threatened the project. Addressing this challenge required innovative solutions, flexibility, and adaptation. Pandemic-related restrictions led to delays in starting the intervention in both sites. On 24th March 2020 a nationwide lockdown was announced for 21 days, which subsequently lasted over two months. At the time the lockdown was announced, the pre-intervention phase was coming to an end and trainings for the intervention phase were being planned in Haryana. The intervention was delayed and could start only by 21st September 2020 in Haryana. In Andhra Pradesh a little over half the population had been screened by ASHAs at the time of the lockdown. Work was resumed in June in areas where lockdown had been eased. In AP the intervention started on 4th December 2020.

After the lockdown eased training for the re-screening and baseline phase needed to be conducted in Andhra Pradesh. Face to face trainings planned earlier were no longer feasible. After several consultations with district health officials, a plan for remote training of ASHAs, following all necessary precautions and guidelines for COVID, was agreed upon. The entire training content for ASHAs was digitised and adapted for remote delivery.

*“Due to the pandemic, it was extremely difficult to conduct the trainings face to face and hence we as a team decided to use online modes for conducting the training. Since our training involved a lot of technological aspects, we developed the online material along with the supervisor and conducted the training of ASHAs and field staff which was received very well for the baseline and ASHA-re screening. (Research Fellow 4, Andhra Pradesh).”*

## Discussion

Operational challenges in the pre-intervention phase of the SMART Mental Health and strategies to overcome these challenges were documented systematically and have been presented in this paper. The SEM provided the analytical framework to analyse these challenges. We found it to be a useful analytical tool as it provided a comprehensive and layered understanding of how contextual challenges that emerge, need to be addressed at multiple levels. The exercise of documenting and systematically reporting challenges provided us with several important lessons.

Some of the operational challenges need to be addressed at the individual level. Issues specific to some AHSAs like poor motivation were addressed through detailed discussions with them. Dissatisfaction with remuneration may emerge as a major challenge in projects that need active participation of community health workers. Financial incentives are an important factor influencing motivation of ASHAs [27, 28]. Similar observations about the role of financial incentives in motivation of community health workers has been made in other LMIC contexts [29]. Remuneration to CHWs is usually provided as fixed salary or incentive against services. It was found that in both cases an ‘expectation gap’ of what was perceived as fair compensation versus what was actually paid, affected motivation of CHWs [29, 30]. Open and transparent dialogue to address remuneration related expectations of ASHAs was an important strategy in the project which helped to address this. Taking time to understand and address specific project related grievances of individual ASHAs was useful in our programme. Relationship and rapport building as well as having both formal and informal communication with ASHAs were important strategies to keep them motivated to be part of the project.

In our context and for several other LMIC context, family, and relatives play an important role in individual decision making [28, 31]. Although research participants and community health workers were our primary stakeholders, we found the need to reach out to their families on several occasions. Interaction with family members may need to be undertaken by a senior supervisory staff who can clearly communicate the project objectives and clarify any doubts of family members. Understanding broader community context and how that affects personal choices should also be taken into consideration while developing implementation research. This has been highlighted in several guidelines for complex interventions [32, 33].

Issues emerging from external institutional level factors like delay in permissions are difficult to address. The ability to obtain government level approvals has flow-on effects downstream. This was a pre-requisite for getting support from PHC doctors and ASHAs to participate in our project. Some of this was facilitated by our continuing relationship with key officials and at other times it just needed patience and repeated discussions with officials. Bureaucracy and research governance have been highlighted as important barriers in undertaking clinical as well as intervention trials in other contexts [34–37]. Some studies have reported need for approvals from multiple agencies increasing bureaucratic requirements from researchers as important barriers. This has been seen to cause delays and increased costs in implementing trials [37, 38]. There have been calls for simplifying research governance with a view to balancing patient safety and research needs, primarily from high income country contexts [34, 36, 37]. This is equally relevant in the Indian context.

At the internal organisational level, it is important to create feedback loops to make continuous improvements where needed. This is crucial for community based mHealth interventions. In recent years there has been a rise in mHealth intervention in LMIC context [39]. However, challenges to scale-up, adoption and integration in routine clinical practice remain. Resource constraints constitute an important barrier [39]. Other barriers may be at the design level. Poor acceptability due to low ease of use, high design complexity and design mismatch with the real-world context of the end users are some challenges reported for mHealth interventions [13, 40, 41]. Agile software development and user centred design have been demonstrated as useful approaches to improve acceptability of mHealth platforms in recent implementation research literature [40, 42–44]. The Agile principles were developed in the field of information sciences and emphasize on using cross-functional teams and engagement with end users to make continuous improvements to software [45]. User centred design principles advocate keeping the end user in the centre of the design process and creating or modifying design to suit end user needs [43]. We found having a dedicated IT team which was updated on a weekly basis about any challenges faced was an important strategy for smooth functioning of technology related components. Continuous adaptations were made based on feedback from the project team, to improve user interface and minimise errors. Interdisciplinary teams consisting IT experts and health professionals, and continuous communication and sharing of ideas between them have been effective strategies for design and implementation of mHealth interventions [46].

Community level interventions need sustained efforts to have buy-in from the community for the project. This is a continuous process and is influenced by community perceptions of research activities that had previously been conducted in the regions. At the very start of the project it is important to reach out to important community leaders. In case of mistrust or opposition from sections of the community we found leveraging the support of influential persons in the community to be most helpful. Engagement with community and religious leaders for wider acceptance of health interventions has been demonstrated to work in several LMIC contexts. Successful engagement with Muslim opinion leaders for improved uptake of maternal and child health services in Nigeria [47], engagement with community leaders in Guinea during the Ebola outbreak [48] and partnership with community leaders in Democratic Republic of Congo for better access to contraceptive and abortion care [49] have been reported. Due to their position in the community, they are in a unique position to positively influence community level health interventions. Our project does not have a formal partnership with community leaders. However regular communication and rapport building has been a helpful strategy to solicit their support for our project.

Since RCTs have strict protocols and need several rounds of data collection in the community, it is important to discuss this comprehensively at the outset. Despite doing this, we found there was also a need for ongoing discussion to explain project objectives and clarify any doubts as and when they emerge from within the community. Stigma associated with mental health adds another level of complexity and can led to reluctance to share data as seen in our case. This underscores the critical importance of integrating stigma reduction activities in any community based mental health intervention [50]. We have also seen in our previous work that there is greater community acceptance of the project with time as benefits from the intervention start reaching community members [51].

Challenges emerging at the policy or external environment may have important implications for implementors. Policies that may not be directly related to our work may impact it in unexpected ways. The citizenship legislations and the ensuing protests and mistrust impacted our ability to collect data in certain communities. It needed community level solutions like leveraging support of key community leaders that had to be adopted.

The COVID-19 pandemic has impacted research trials across the globe. Management of ongoing trials has faced several challenges and some trials have been temporarily paused [52]. Some of the challenges include difficulty in recruitment and obtaining informed consent from participants, conducting training and data collection. Communication with staff and stakeholders and onsite monitoring have also been impacted due to the pandemic [53]. The COVID-19 pandemic disrupted the ‘when and how’ which were planned for certain timely deliverables of our project. As a result, intervention delivery was delayed in both sites. Since this was a community-based trial, COVID related restrictions led to several barriers in undertaking project activities. Flexibility and making adaptations may be needed in such cases. We had to find solutions like remote training of community health workers, remote monitoring of field staff, adjusting timelines and seeking approval to make certain protocol amendments. Recent literature supports this view. Adaptation and flexibility in trial management has been crucial in most cases to overcome challenges posed by the pandemic. A study based on inputs from trial managers from UK found that sponsors eased approval for use of digital tools for recruitment and consent in some cases. Use of technology to undertake activities remotely has emerged as an important solution. Both electronic and digital communication tools to undertake research have been used for participant recruitment, obtaining informed consent, providing health services remotely and substituting for onsite monitoring and communication with staff [53, 54]. There is need to further document and better understand strategies being adopted by researchers to deal with barriers emerging because of the pandemic.

### Implications of the findings

Systematic documentation of operational challenges in executing pre-intervention research activities, has been a valuable exercise for the project. It has provided important insights for the project team on critical factors that might impact implementation fidelity. A similar exercise will be undertaken during the other study phases to identify other operational challenges and strategies adopted to overcome them.

The findings potentially have implications beyond this project. It may be of relevance to implementation of complex health service strengthening strategies more broadly and contribute to the literature on challenges of operationalising such interventions in LMIC settings.

### Limitations

The paper uses insights of research fellows, the IT staff and project supervisors to understand operational challenges faced during implementation of the project. However, field supervisors and field investigators were not formally interviewed as senior staff were able to provide information on field level operational challenges. Although the lead author attended some meetings with field staff, this was done on an ad hoc basis and may not have systematically captured their perspectives. Field visits could only be made to the Haryana site as COVID related travel restrictions prevented visits to the Andhra Pradesh sites. In focussing mainly on project implementer views, this may introduce some bias as other perspectives were not prioritised. The paper is not intended as an unbiased evaluative piece but as a reflective piece on the research practice from those directly engaged in implementation of the intervention.

## Conclusion

Implementation of complex interventions may pose challenges at several levels. Community-based interventions in LMIC contexts have additional layers of complexity that have not been well documented. Identification of challenges in the pre-trial phase and developing strategies to address these has generated important lessons which may be of value for future health service intervention research in LMIC contexts. We recommend: (1) the need to engage with all stakeholders continually and actively during the implementation as well design process to have greater buy-in for the research; (2) a high degree of agility in making adaptations and leveraging technology to overcome the major and unanticipated barriers related to COVID; (3) a need to rigorously document challenges and adaptations to aid with the interpretation of findings from process evaluations and (4) policy reform to support more streamlined research governance processes.

## Data Availability

Not applicable.

## Abbreviations

ANM: Auxiliary nurse midwife
ASHA: Accredited Social Health Activist
CMD: Common mental disorders
cRCT: Cluster randomized control trial
COVID-19: Coronavirus disease 2019
EDSS: Electronic decision support system
IT: Information technology
LMICs: Low and middle-income countries
mHealth: Mobile health
mhGAP: Mental Health Gap Action Programme
PHC: Primary health centre
PWLE: Person with lived experience
SEM: Socio-ecological Model
SMART: Systematic Medical Appraisal Referral and Treatment
